# Supplementary material for: How to promote the quality of technical education? A fuzzy-set qualitative analysis of 16 technical colleges in China
Source: PLoS One. 2025 Oct 6;20(10):e0332115. doi: 10.1371/journal.pone.0332115 (PMC12500140; doi:10.1371/journal.pone.0332115)
Supplement: Attachment — Submitted filename: PlosOne_comments_25_07768.pdf [file pone.0332115.s002.pdf]

Dear authors,

Thanks for providing me the opportunity to read your ongoing work. The study conducts a fuzzy qualitative analysis of technician colleges in China trying to assess the inter-dependecies influencing various factors for quality education. Below, I enumerate my comments that I had while reading your analysis:

Main comments

1. Unclear Research Question: Given that this is a qualitative paper, it is especially important to clearly articulate the underlying economics and motivation for the study. The introduction does not adequately explain the broad research question or why this topic merits investigation. Even in the later sections, the study's objective remains unclear.

2. There is a repeated paragraph within the "Sample Data and Collection" section. The strategy for sample collection is described twice, which should be streamlined for clarity and conciseness.

3. The construction of antecedent variables is presented with little to no supporting citations. It is unclear what theoretical or empirical foundations guided the selection and construction of these variables.

4. The authors reference the CIPP model under the theoretical framework but provide minimal explanation of what the model entails. Additionally, there is a lack of citations or references to support the use of this framework.

5. Issues in the Empirical Section: The empirical section does not clearly explain how the scores were obtained. Although the authors claim causal relationships, they do not present convincing arguments or methodological rigor to support these claims. Furthermore, no justification is provided for the thresholds used in the configuration analysis.
