# Supplementary material for: How to promote the quality of technical education? A fuzzy-set qualitative analysis of 16 technical colleges in China
Source: PLoS One. 2025 Oct 6;20(10):e0332115. doi: 10.1371/journal.pone.0332115 (PMC12500140; doi:10.1371/journal.pone.0332115)
Supplement: Attachment — Submitted filename: Comments -PONE-D-25-07768.docx [file pone.0332115.s003.docx]

**COMMENTS FOR MANUSCRIPT PONE-D-25-07768**

1. The study presents the results of original research.

Yes

2. Results reported have not been published elsewhere.

To the best of my knowledge, the results in the report have not been published elsewhere.

3. Experiments, statistics, and other analyses are performed to a high technical standard and are described in sufficient detail.

The sampling method is not comprehensively described. Why are they choosing only high performing colleges? There is also no justification on the number of colleges included in the study. It is also not clear why they selected the number of selected colleges differs across regions (for example, why did they choose 5 in the east, 2 in the middle, 6 in the west and so on?).

4. Conclusions are presented in an appropriate fashion and are supported by the data.

Paragraph 2 of the conclusion is supposed to be part of the discussion section, which is completely absent in the paper.

5. The article is presented in an intelligible fashion and is written in Standard English.

The language used in the article is flawless, but will need some proof reading to remove the minor grammatical errors currently in the manuscript (for example, the first sentence of the theoretical model has a some missing words). However, there is a lot of repetitions in the sampling section.

6. The research meets all applicable standards for the ethics of experimentation and research integrity.

Yes.

7. The article adheres to appropriate reporting guidelines and community standards for data availability.

Yes.
