# Supplementary material for: How to promote the quality of technical education? A fuzzy-set qualitative analysis of 16 technical colleges in China
Source: PLoS One. 2025 Oct 6;20(10):e0332115. doi: 10.1371/journal.pone.0332115 (PMC12500140; doi:10.1371/journal.pone.0332115)
Supplement: Attachment — Submitted filename: Response to Reviewers.docx [file pone.0332115.s006.docx]

Rebuttal letter

Dear editors and reviewers,

Thank you for giving us the opportunity to submit a revised draft of the manuscript “How to Promote the Quality of Technical Education？A Fuzzy-set Qualitative Analysis of 16 Technical Colleges in China” for publication in the Journal of PLOS ONE. We appreciate the time and effort that you and the reviewers dedicated to providing feedback on our manuscript and are grateful for the insightful comments on and valuable improvements to our paper.

We feel great thanks for your professional review work on our article. As you are concerned, there are several problems that need to be addressed. According to your nice suggestions, we have made extensive corrections to our previous draft, the detailed corrections are listed below.

We sincerely thank the editor and all reviewers for their valuable feedback that we have used to improve the quality of our manuscript. The reviewer comments are laid out below in italicized font and specific concerns have been numbered. Our response is given in normal font and changes. The following are separate responses to academic editors and reviewers, corresponding to each opinion in the email. We believe that these revisions have effectively addressed all your concerns and greatly improved the overall quality and rigor of our manuscript. We welcome any further suggestions or comments you may have and are committed to making additional improvements if necessary.

Best regards,

Fan Zhang

Department of Education/Shaanxi Normal University/Xi’an/Shaanxi Province/China

**Reply to comments from academic editors**

Reviewer #1

| 1. I suggest authors expand the justification by clearly highlighting the relevance and original contribution of the research in relation to previous studies. | Thank you very much for your valuable suggestions. We have added explanations about technical education or technical colleges in the article to clarify the background of the topic selection and its connection with previous studies. |
| --- | --- |
| 2. In the literature review section, authors should avoid unnecessary repetition and better integrate national and international studies into clear topics. Don't forget to correct incomplete or incorrect references. | Sincerely thank you for your comment. We have revised the literature review section, organizing and summarizing the current research status both at home and abroad, and introducing the research questions of this paper. |
| 3. It may be necessary to reduce narrative descriptions of cases, focusing only on key points and how they exemplify the configurations found. | Thank you very much for your valuable suggestions. We have removed the case description and instead have moved it to the discussion section to provide a more in-depth exploration of the practical applications of these configurations. |
| 4. Authors should expand on the study's practical contributions. | Thank you for your feedback. We further elaborated on the significance, value and contributions of this study. |

Reviewer #2

| 1. From the introduction onwards, the manuscript suffers from a disorganized structure and weak logical flow. The argument is difficult to follow, and the narrative lacks a coherent progression typical of academic writing. | Thank you for your suggestion. By studying the literature, we reorganized the framework of the article, optimized the structure, strengthened the logical connections, and have now effectively resolved the problems of structural disorder and logical incompleteness. |
| --- | --- |
| 2. The topic, while generally relevant, is poorly framed and lacks originality. The research findings do not provide novel insights or actionable implications for advancing the field of technical education in China or internationally. | Thank you very much for your valuable suggestions. We have refined the research framework and based on the results of this study, we have put forward suggestions for technical education in China and around the world. |
| 3. The literature review appears to be a mere aggregation of prior studies without synthesis or critical evaluation. It fails to establish a solid theoretical foundation for the study. | Sincerely thank you for your comment. We have revised the literature review section, organizing and summarizing the current research status both at home and abroad, and introducing the research questions of this paper. |
| 4. The use of fuzzy-set qualitative comparative analysis (fsQCA) is inadequately explained and poorly executed. The sampling rationale, variable selection, and calibration process lack transparency and rigor, which raises serious concerns about the validity of the results. | Thank you very much for your valuable suggestions. We re-explained the method of fsQCA, its advantages and limitations, and provided an application explanation in the context of educational quality issues. |
| 5. The results are presented as mechanical outputs of the fsQCA software, with minimal interpretation or theoretical reflection. The discussion does not go beyond descriptive repetition of configurations and fails to provide meaningful insights. | Thank you very much for your valuable suggestions. We recognize that in the initial draft, the interpretation and theoretical discussion of the fsQCA results were indeed not sufficiently in-depth, resulting in the discussion section appearing overly mechanical and descriptive. In the revised version, we have comprehensively reorganized and expanded the results section, adding detailed explanations and theoretical analyses of the fsQCA outputs. Specifically:  Result Explanation: We conducted in-depth theoretical analyses for each configuration path, explaining why these specific condition combinations lead to high-quality or non-high-quality development outcomes. We combined relevant theoretical frameworks, such as the social service function theory and the regional economic development theory, to support our explanations.  Discussion Deepening: In the discussion section, we not only described the configuration paths but also explored the mechanisms and logic behind these paths. For instance, we analyzed the core role of social service functions in the high-quality development of technical colleges and further illustrated these mechanisms through specific cases (such as Shanghai Senior Technical College and Beijing Industrial Technician College).  Theoretical Connection: We further connected the results with existing literature theories, exploring how our findings expand or challenge existing theoretical viewpoints. This not only enhanced the theoretical depth of the research but also provided new perspectives for subsequent studies. |
| 6. The reference formatting is inconsistent throughout the manuscript, with noticeable citation errors. This indicates a lack of attention to academic standards and detracts from the manuscript’s credibility. | Thank you for your suggestion. We have checked and corrected the reference format of the article, thereby enhancing its rigor. |

Reviewer #3

| 1. While the introduction provides a comprehensive overview of the topic and relevant policy context, the specific academic contribution of the study remains unclear and should be more explicitly articulated. In my opinion the contribution is the weakest point of the paper. | Thank you for your feedback. We further elaborated on the significance, value and contributions of this study. |
| --- | --- |
| 2. The literature review, while comprehensive in scope, is largely descriptive and lacks the critical engagement and analytical depth necessary to meaningfully support the research. It reads more like a background summary than a foundation for an original academic contribution. To strengthen this section, the authors should critically synthesize the reviewed studies, identify gaps in the existing literature, and clearly formulate research hypotheses that articulate the study's intended scientific contribution. | Sincerely thank you for your comment. We have revised the literature review section, organizing and summarizing the current research status both at home and abroad, and introducing the research questions of this paper. |
| 3. The research design is generally conceptually relevant, but it requires a clearer and more systematic structure. The chosen methodology lacks sufficient justification, and alternative methodological approaches are not discussed, nor are their respective advantages and limitations. This omission weakens the analytical rigor and should be addressed as part of a more comprehensive robustness analysis, which is currently underdeveloped. | Thank you very much for your valuable suggestions. The research constructed an index system based on the triple helix structure, and re-explained the comparison between QCA and fsQCA methods, exploring their respective advantages and limitations. |
| 4. While the paper proposes several relevant directions for policy, the recommendations remain overly general and would benefit from clearer prioritization, contextual differentiation, and discussion of feasibility and implementation mechanisms. | We agree with the reviewer's point that the policy recommendations section needs to be more specific and in-depth. In the revised version, we have made comprehensive improvements to the policy recommendations section to ensure that the suggestions are more operational and targeted:  Clarifying priorities: We have prioritized the proposed policy recommendations and identified which measures should be implemented first. For example, we emphasized the importance of strengthening social service functions and improving students' skill levels, and explained why these measures should be regarded as priorities.  Analysis of background differences: We have added a detailed analysis of the differences in background among different regions, discussing the differences in economic development levels, industrial structures, and educational resources among the eastern, central, western, and northeastern regions, and proposed specific policy recommendations tailored to these differences. |

Reviewer #4

| 1. Unclear Research Question: Given that this is a qualitative paper, it is especially important to clearly articulate the underlying economics and motivation for the study. The introduction does not adequately explain the broad research question or why this topic merits investigation. Even in the later sections, the study’s objective remains unclear. | Thank you for your feedback. We have revised the introduction section, clearly presenting the research question and its related content, as well as the significance and value of the study. Additionally, the thresholds for configurational analysis have been explicitly supplemented in the text. |
| --- | --- |
| 2. There is a repeated paragraph within the ”Sample Data and Collection” section. The strategy for sample collection is described twice, which should be streamlined for clarity and conciseness. | Thank you very much for your valuable suggestions. We have removed the duplicate parts and reorganized the description of the sample collection process. |
| 3. The construction of antecedent variables is presented with little to no supporting citations. It is unclear what theoretical or empirical foundations guided the selection and construction of these variables. | We take your comments very seriously. We have provided literature support in the construction part of the pre-defined variables, referring to the relevant studies of aa (2020) and bb (2021), which has further strengthened the theoretical basis. |
| 4. The authors reference the CIPP model under the theoretical framework but provide minimal explanation of what the model entails. Additionally, there is a lack of citations or references to support the use of this framework. | Thank you for your suggestion. We have revised the research framework of the article. The research is based on a three-helix structure to build an index system, which enhances its scientific nature. |
| 5. Issues in the Empirical Section: The empirical section does not clearly explain how the scores were obtained. Although the authors claim causal relationships, they do not present  convincing arguments or methodological rigor to support these claims. Furthermore, no justification is provided for the thresholds used in the configuration analysis. | Thank you for your feedback. We have revised the introduction section, clearly presenting the research question and its related content, as well as the significance and value of the study. Additionally, the thresholds for configurational analysis have been explicitly supplemented in the text. |

Reviewer #5

| 1. The sample for analysis includes 16 technician colleges selected from a list of 40 colleges included in a book profiling “outstanding technician colleges.” Why were colleges selected from this book? If the intent is to identify configurations related to high quality, it seems strange to start from a sample of “outstanding” colleges, which limits the ability to distinguish high quality from low quality since they are all seemingly exceptional. Why don’t the authors use a sample that is representative of all colleges? | Thank you for your comment. We have reorganized the description of the sample collection process as per the requirements. The cases in this book are selected from 40 outstanding technical colleges, but only a portion of them passed the government's high-quality assessment. Therefore, I will define the "high-level" technical colleges that passed the government assessment as truly high-quality, which is "1", while the other excellent schools that failed the review will be classified as "0". I hope to conduct research on these 40 schools based on the index construction, and then use fuzzy set qualitative analysis to find the way to achieve even better results. However, during the data collection process, only the data of 16 schools were complete. Therefore, I will use these 16 technical colleges as samples for calculation. |
| --- | --- |
| 2. The process for selecting the 16 colleges for analysis out of the population of 40 colleges is not clear. At first, it sounds like they are randomly selected after stratifying them into four regions. However, at the end of page 3, the authors say “After further data collection, 16 technician colleges were selected as the final research objects,” How were these 16 selected, what was the further data collection and how was it used to select them? | Thank you for your comment. We have reorganized the description of the sample collection process as per the requirements. |
| 3. The last paragraph in the section “(1) Sample and Data Collection” appears to duplicate the information in the preceding paragraphs. | Thank you very much for your valuable suggestions. We have removed the duplicate parts and reorganized the description of the sample collection process. |
| 4. I think the authors should refrain from using language that implies causality. It does not seem like this method—combined with the small sample with issues of representativeness described above—can make causal inferences. Instead, configurations/characteristics should be described as associated with high quality. | Thank you for your suggestion. We have revised the language expression of the article. |
| 5. The description of the construction of antecedent variable indicators is confusing. It is not always clear how they are constructed, particularly for the indicators that are described as being in “minutes” in table 2. What does “minutes” mean here, and how are the points systems described in various rows of table 2 developed? | We take your comments very seriously. We have added literature support in the construction part of the pre-existing variables, further strengthening the theoretical foundation and re-optimizing the elaboration of the indicator construction. |
| 6. I don’t understand the difference between “observation index” and “indicator” in table 2. It seems like observation index includes indicators, except when they need to be defined with some additional points system, which is then described in the indicator column. | Thank you for your comment. We have reworked and refined the explanation of the indicator construction. |
| 7. Many of the indicators are basically just proxies for the size of the college (e.g., number of students, number of teachers) or are highly influenced by size (e.g., number of papers published). Wouldn’t it be better to have indicators that are normalized by size, like a student-teacher ratio? | Thank you for your comment. We have reworked and refined the explanation of the indicator construction. |
| 8. Instead of using “human” as a unit in table 2, “persons” or “individuals” might be a better term. | Thank you for your comment. We have reworked and refined the explanation of the indicator construction. |
| 9. It is not clear how the binary outcome is constructed. Page 8 describes many indicators of high quality, but I do not understand how they are then translated into a binary indicator. | Thank you for raising this important concern. We appreciate your careful review of our manuscript. In response to your question regarding the construction of the binary outcome, we have thoroughly reorganized and clarified this aspect in the revised version.  We have now provided a detailed explanation of how the binary outcome is constructed. Specifically, we synthesized multiple high-quality indicators applied a clear decision rule to convert them into a binary variable. As presented in the newly organized Table 4, we first identified the key criteria for high-quality development based on the indicators such as student skill levels, teacher qualifications, research outputs, industry-education integration, and social service contributions. Then, if a technical vocational college met or exceeded the pre-defined thresholds for these comprehensive criteria, it was classified as having "high-quality development" (coded as 1); otherwise, it was categorized as "non-high-quality development" (coded as 0). This table now systematically outlines the specific indicators, their respective weights (if applicable), and the thresholds used in the binary classification process, ensuring transparency and clarity in our methodology. |

Reviewer #6

| 1. The introduction section of the article does not raise a scientifically meaningful question. The research lacks a central theme and focused research content. | Thank you for your feedback. We have revised the introduction section and clearly expounded the research question and its related contents. |
| --- | --- |
| 2. The data in this study comes from secondary sources, specifically from a book. The sampling process and the characteristics of the sampled data are not described in detail, making it impossible to verify the authenticity and reliability of the data. Additionally, the overall workload of the study is clearly insufficient. | Thank you for your comment. We have reorganized the description of the sample collection process as per the requirements. |
| 3. The selection of the indicator system lacks scientific rigor and is based on the author’s subjective judgment. Since the paper does not have a core research question, the author’s choice of indicators is arbitrary, rendering the evaluation scientifically unfounded. Moreover, the indicators exhibit endogeneity (interdependence), which is not thoroughly explained. | Thank you for your suggestion. We have revised the research framework of the article. The research is based on a three-helix structure to build an index system, which enhances its scientific nature. |
| 4. The empirical analysis is overly generalized and does not address the actual problem. It merely follows a routine evaluation method without exploring the formation mechanism of the scientific issue, causal analysis, or scientific pathways. This paper is merely a subjective indicator evaluation study without substantive theoretical or empirical contributions. | Thank you for your incisive and constructive feedback. We deeply appreciate your critical review, which has pointed out key areas for improvement in our study. First, to address the issue of overly generalized empirical analysis, we have integrated the CIPP (Context, Input, Process, Product) model and the Triple Helix theory into our research framework. The CIPP model provides a comprehensive perspective to evaluate the educational quality of technical vocational colleges from multiple dimensions, including the context in which education occurs, the resources inputted, the teaching and learning processes, and the final educational products. The Triple Helix theory, on the other hand, emphasizes the interactions and collaborations among government, colleges, and enterprises, which are crucial for understanding the complex ecosystem of technical vocational education. By incorporating these two theoretical models, we have re - structured our empirical analysis to explore the underlying mechanisms that drive the development of technical vocational colleges, rather than simply conducting a surface - level evaluation. |

Reviewer #7

| 1. General comment: I have significant doubts about the value added of the paper. After reading it I don’t understand what that value added is as the authors don’t indicate it. Additionally as the paper focuses on China the authors should draw some conclusions for the international audience but that does not happen in the paper. | Thank you very much for your valuable suggestions. We have revised the article and elaborated on the significance and value of the research. Based on the research results, we also put forward suggestions for technical education in the country and globally. |
| --- | --- |
| 2. The abstract is very difficult to understand and should be completely rewritten. | We appreciate your feedback regarding the abstract. We agree that clarity is essential for effective communication of our research. We have thoroughly revised the abstract to enhance its readability and accessibility. |
| 3. The Introduction is very superficial. It lacks an indication what data and method will be used in the paper, what value added the paper has and what its structure is. | We sincerely appreciate your valuable suggestions. We have thoroughly revised the introduction section.  Revised Abstract:  This study focuses on how technical and vocational education and training (TVET) institutions can effectively enhance educational quality to cultivate a globally competitive industrial workforce, addressing the World Bank’s projection that over 1.1 billion jobs worldwide will undergo skill transformations in the next decade due to automation, digitalization, and the green economy transition. Employing the fuzzy-set qualitative comparative analysis (fsQCA) method, the research conducts an in-depth analysis of developmental data from 16 Chinese TVET institutions to identify key factors influencing their competitiveness.  The findings reveal that social service constitutes the foundation of high-quality vocational education. The social service capacity of TVET institutions is primarily reflected in vocational skill training, aligning with the core philosophy of Singapore Polytechnic’s "SkillsFuture" initiative. Through data analysis, a "social service-driven" development mechanism is identified: under similar conditions, TVET institutions achieve high-quality development by participating in government-funded vocational training programs. Simultaneously, two types of developmental bottlenecks are uncovered: (1) the "student skill level-international exchange constraints" type, where limited student proficiency and international collaboration hinder institutional progress; and (2) the "social service-technological R&D constraints" type, where weak social service delivery and technology transfer capabilities act as critical barriers.  The outcomes provide a robust reference for global TVET stakeholders and policymakers to optimize industrial talent cultivation strategies and deepen integration into global value chains. The methodological framework also holds transferability to other domains, enabling regions to pinpoint success pathways and avoid ineffective measures. |
| 4. The Literature Review is superficial too. It is difficult to understand and simply boring. | Sincerely thank you for your comment. We have revised the literature review section, organizing and summarizing the current research status both at home and abroad, and introducing the research questions of this paper. |
| 5. Based on the description in the paper I have no idea why the authors have two parts dedicated to the description of the data sample: one on page 3 (Sample and Data Collection) and one on page 9 (Data Acquisition and Calibration) | Thank you very much for your valuable suggestions. We have removed the duplicate parts and reorganized the description of the sample collection process. |
| 6. Why is the first paragraph on page 4 a copy-paste of the last 4 paragraphs on the previous page? | Thank you for your suggestion. We have revised the language expression of the article. |
| 7. The authors state on page 4 that they chose 40 cases of outstanding technician colleges. What is the reason behind this? All these all technician colleges in China? Or have the authors applied a procedure to select those 40 colleges? This totally unclear. | Thank you for your comment. We have reorganized the description of the sample collection process as per the requirements. The cases in this book are selected from 40 outstanding technical colleges, but only a portion of them passed the government's high-quality assessment. Therefore, I will define the "high-level" technical colleges that passed the government assessment as truly high-quality, which is "1", while the other excellent schools that failed the review will be classified as "0". I hope to conduct research on these 40 schools based on the index construction, and then use fuzzy set qualitative analysis to find the way to achieve even better results. However, during the data collection process, only the data of 16 schools were complete. Therefore, I will use these 16 technical colleges as samples for calculation. |
| 8. The section Construction of antecedent variable indicators has to be completely rewritten. Most of its content can probably be presented in a Table. | Thank you for your comment. We have reworked and refined the explanation of the indicator construction. |
| 9. The sections following the Construction of antecedent variable indicators section are difficult to understand and difficult to follow. I have no idea what the authors are trying to describe in those section. | Thank you for your comment. We have reworked and refined the explanation of the indicator construction. |

Reviewer #8

| 1. The sampling method is not comprehensively described. Why are they choosing only high-performing colleges? There is also no justification on the number of colleges included in the study. It is also not clear why they selected the number of selected colleges differs across regions (for example, why did they choose 5 in the east, 2 in the middle, 6 in the west and so on?). | Thank you for your comment. We have reorganized the description of the sample collection process as per the requirements. The cases in this book are selected from 40 outstanding technical colleges, but only a portion of them passed the government's high-quality assessment. Therefore, I will define the "high-level" technical colleges that passed the government assessment as truly high-quality, which is "1", while the other excellent schools that failed the review will be classified as "0". I hope to conduct research on these 40 schools based on the index construction, and then use fuzzy set qualitative analysis to find the way to achieve even better results. However, during the data collection process, only the data of 16 schools were complete. Therefore, I will use these 16 technical colleges as samples for calculation. |
| --- | --- |
| 2. Paragraph 2 of the conclusion is supposed to be part of the discussion section, which is completely absent in the paper. | Thank you for your feedback. We are aware that there were indeed issues with the chapter structure in the initial draft, which led to some content being misplaced in the conclusion section. In the revised version, we have adjusted the structure of the paper to ensure that each part's content aligns with its intended function:  Structural adjustment: We moved the content involving in-depth analysis and explanation in the conclusion section to the discussion section, and expanded and enriched the discussion section to make it an independent and complete chapter.  Content optimization: We ensured that the discussion section not only includes detailed explanations and theoretical discussions of the research results, but also covers the background analysis and implementation mechanisms of policy recommendations. Thus, the discussion section can comprehensively present the depth and breadth of the research, while the conclusion section focuses on summarizing the main findings and contributions of the study. |
| 3. The language used in the article is flawless but will need some proof reading to remove the minor grammatical errors currently in the manuscript (for example, the first sentence of the theoretical model has some missing words). However, there is a lot of repetitions in the sampling section. | Thank you for your suggestion. We have proofread the article and resolved the minor grammatical errors present in the current manuscript. |
