# Supplementary material for: How to promote the quality of technical education? A fuzzy-set qualitative analysis of 16 technical colleges in China
Source: PLoS One. 2025 Oct 6;20(10):e0332115. doi: 10.1371/journal.pone.0332115 (PMC12500140; doi:10.1371/journal.pone.0332115)
Supplement: Attachment — Submitted filename: Comments -PONE-D-25-07768_R1.docx [file pone.0332115.s005.docx]

**COMMENTS FOR MANUSCRIPT PONE-D-25-07768_R1**

1. The study presents the results of original research.

Yes.

2. Results reported have not been published elsewhere.

To the best of my knowledge, the results in the report have not been published elsewhere.

3. Experiments, statistics, and other analyses are performed to a high technical standard and are described in sufficient detail.

Comments raised on the clarity of the sampling have been addressed.

4. Conclusions are presented in an appropriate fashion and are supported by the data.

Yes.

5. The article is presented in an intelligible fashion and is written in Standard English.

Yes.

6. The research meets all applicable standards for the ethics of experimentation and research integrity.

Yes.

7. The article adheres to appropriate reporting guidelines and community standards for data availability.

Yes.
