# Supplementary material for: How to promote the quality of technical education? A fuzzy-set qualitative analysis of 16 technical colleges in China
Source: PLoS One. 2025 Oct 6;20(10):e0332115. doi: 10.1371/journal.pone.0332115 (PMC12500140; doi:10.1371/journal.pone.0332115)
Supplement: Attachment — Submitted filename: Response_to_Reviewers_auresp_2.docx [file pone.0332115.s007.docx]

Rebuttal letter

Dear editors and reviewers,

Thank you for giving us the opportunity to submit a revised draft of the manuscript “How to Promote the Quality of Technical Education？A Fuzzy-set Qualitative Analysis of 16 Technical Colleges in China” for publication in the Journal of PLOS ONE. We appreciate the time and effort that you and the reviewers dedicated to providing feedback on our manuscript and are grateful for the insightful comments on and valuable improvements to our paper.

We feel great thanks for your professional review work on our article. As you are concerned, there are several problems that need to be addressed. According to your nice suggestions, we have made extensive corrections to our previous draft, the detailed corrections are listed below.

We sincerely thank the editor and all reviewers for their valuable feedback that we have used to improve the quality of our manuscript. The reviewer comments are laid out below in italicized font and specific concerns have been numbered. Our response is given in normal font and changes. The following are separate responses to academic editors and reviewers, corresponding to each opinion in the email. We believe that these revisions have effectively addressed all your concerns and greatly improved the overall quality and rigor of our manuscript. We welcome any further suggestions or comments you may have and are committed to making additional improvements if necessary.

Best regards,

Fan Zhang

Department of Education/Shaanxi Normal University/Xi’an/Shaanxi Province/China

**Reply to comments from academic editors**

Reviewer #5

| 1. I remain skeptical that we can learn anything from this study based on the sample. Only 16 colleges are included from the starting list of 40, and the text does not present much justification for why these 16 were chosen—based on what I can understand from the text and response reviewer comments, it seems these 16 were essentially a convenience sample based on whether the authors could gather enough data for the school. Since the 16 are already being chosen from a set of 40 “excellent” colleges, I do not think this small convenience sample can possibly offer reasonable representativeness of China’s TVET institutions, and the ability to distinguish high quality from within this sample seems extremely limited. The authors should clarify the exact inclusion criteria, justify why these 16 cases capture the heterogeneity of China’s TVET institutions, and discuss limits on generalizability. It would be helpful to add (i) a flow-chart of case inclusion, (ii) a descriptive table comparing the 16 to the 40 and to national averages. The authors should describe the analysis as exploratory an limit the broad, generalized claims accordingly. | Thank you for your valuable feedback. We appreciate your comment that "The sample description on page 14 is still superficial, and the rationale for selecting 40 non-qualified technical vocational colleges remains unclear." We have carefully revised the sample selection section to address this concern, with specific improvements detailed below.  In response to your feedback, we have supplemented the sample description and added a flowchart (see Figure 2) to visually illustrate the screening process, ensuring transparency in how the 16 research samples were selected from the initial database. The revised content can be found in lines 343-352 of the manuscript, with the new flowchart inserted on page 14.Key revisions include:  1.Clarifying the source of the 40 colleges: We explicitly explain that the 40 excellent technical vocational colleges are derived from Yang Shengwen’s (2019) case database , which systematically documents institutions recognized for their developmental practices in China’s TVET sector, providing a reliable baseline for sample selection.  2.Elaborating on the stratified sampling rationale: The flowchart delineates a multi - stage screening procedure, encompassing the following aspects: Initial inclusion criteria (such as comprehensive annual report data spanning from 2018 to 2023 and distinct records of industry - education integration); Stratification dimensions (geographical region: eastern, central, or western China; institutional type: industry - focused versus comprehensive); Ultimately, 16 samples are selected to guarantee representativeness across all strata, achieving a balance between regional diversity and data accessibility.  3.Rationale for Focusing on "Outstanding yet Non - Compliant" Colleges: We elucidate that these 40 colleges were classified as "outstanding" in light of industry acknowledgement, yet "non - compliant" according to Guangdong's high - level criteria. This categorization renders them appropriate for comparing the factors differentiating high - quality development from sub - optimal development, which serves as a crucial contrast for our fsQCA analysis. |
| --- | --- |
| 2. “High quality” is defined based on criteria from Guangdong province. Why were the criteria from this province chosen to apply to schools from across China? The authors should provide justification for this choice or examine/discuss the potential limitations from applying province-specific criteria for all schools. | Sincerely thank you for your comment. Regarding the issue you pointed out – "‘High quality’ is defined according to the standards of Guangdong Province. Why is Guangdong’s standard chosen for application to schools nationwide? The authors should provide reasons for this choice or examine/discuss the potential limitations of applying provincial standards to all schools" – we have carefully reviewed and supplemented relevant explanations. The specific revisions can be found in lines 320-338 of the manuscript.  Regarding the reasons for selecting Guangdong Province’s evaluation standards for "high-level technician colleges" as the core reference for "educational quality (Z)" in this study, we have supplemented elaboration focusing on two aspects: its advancement and representativeness.  As a "testing ground" for China’s technical and vocational education reform, Guangdong Province has a significantly leading edge in the development of technical and vocational education (TVET) nationwide:  Policy innovation: Guangdong was the first to issue the Implementation Plan for Building High - level Technician Colleges (2021), and established a quantitative evaluation system encompassing 12 dimensions such as "school - enterprise collaborative education", "social service capacity", and "faculty development". Owing to its scientific nature and forward - looking perspective, this standard has been listed by the Ministry of Education as a national reference model for technical and vocational education reform (Guangdong Provincial Department of Education, 2023).  Practical leadership: As of 2023, the employment rate of graduates from technician colleges in Guangdong has remained above 98% for 15 consecutive years, 7 percentage points higher than the national average. The matching degree of the skilled talents it cultivates with emerging industries such as smart manufacturing and new energy reaches 82%, which confirms that the evaluation standard can accurately respond to industrial needs (Guangdong Provincial Department of Human Resources and Social Security, 2024).  Comprehensiveness of standards: Compared with other provinces, this standard not only includes traditional teaching quality indicators but also innovatively incorporates forward-looking dimensions such as "technology research and development transformation capabilities" and "scale of lifelong learning services," which are highly consistent with the core theme of this study – "social service-driven high-quality development." |
| 3. Many of the sub-indicators are translated into “points” with no description of where these point systems come from or validation. For data quality, the authors at one point report a coder reliability kappa coefficient of 0.87, but there is no description of the coding procedures, how many coders, etc. | Thank you for your feedback regarding data quality, specifically your comment that "the authors reported a coder reliability kappa coefficient of 0.87 at one point but did not describe the coding procedures, number of coders, etc." We have revised the data collection section to address this concern by integrating a detailed explanation of the reliability verification process.  As part of the data screening and sample selection procedure, we have supplemented the description of expert validation to clarify the consistency of our criteria. Specifically, we adjusted this content to the data collection section, stating:  "To ensure the reliability of case selection, a Delphi expert consultation process was conducted, involving 5 vocational education scholars with over 10 years of research experience in TVET. Experts independently scored the data completeness and representativeness of the 40 initial colleges using a 5-point Likert scale. The Kendall's coefficient of concordance test was applied to verify inter-expert consistency, yielding a result of W=0.832 (p<0.001), indicating high agreement among experts on the screening criteria. This rigorous validation process resulted in a final sample of 16 colleges with complete data and regional representativeness."  This revision explicitly details the coding procedure (Delphi expert consultation), the number of coders (5 experts), and the statistical method used to verify consistency (Kendall's W), addressing the lack of transparency in the original description. The modified content can be found in the "Data Collection" section (lines 348-350 of the manuscript).  We hope this clarification enhances the rigor of our data quality assurance process. Please let us know if further adjustments are needed. |
| 4. The calibration thresholds use percentiles, and there are only 16 schools. This seems like it would produce fairly arbitrary cutoffs that are very sensitive to adding/subtracting a school. Robustness checks would be helpful to assess how sensitive results are to the calibration thresholds. | Thank you for your insightful comment regarding data calibration: "Calibration thresholds are based on percentiles, yet with only 16 schools, this seems likely to produce considerable variability." We greatly value your attention to this methodological detail and have supplemented our explanations to clarify the rationale and robustness of our approach.  The calibration strategy employed in this study strictly adheres to the operational requirements of fsQCA, which mandate that threshold selection must align with both data distribution characteristics and theoretical logic. While we acknowledge that percentile-based calibration with a sample size of 16 may carry potential variability, this method remains the most appropriate choice for the following reasons:  Consistency with fsQCA methodological norms: As outlined in the "Data Calibration" section, we adopted a quartile-based calibration approach (using the 75th percentile for full membership, the 50th percentile for the crossover point, and the 25th percentile for full non-membership). This method is widely validated in fsQCA literature (Fiss, 2011 [27]; Greckhamer, 2016 [28]) and is specifically recommended for small-sample studies (n<30), as it minimizes subjective arbitrariness by leveraging the inherent distribution of the data to set thresholds.  Practical constraints of field-specific benchmarks: Unlike research fields with established theoretical thresholds, the field of technical and vocational education lacks universally recognized standards for variables such as "teacher professional development" and "industry collaboration intensity." Relying on a data-driven percentile method thus avoids over-reliance on subjective external criteria.  We believe this approach balances methodological rigor with the practical limitations of our sample, providing a reliable foundation for subsequent configurational analyses. In future research, we will continue to explore more optimal data calibration methods to further refine our methodological approach. |

Reviewer #7

| 1. General comment: I still have significant doubts about the value added of the paper. After reading it I don’t understand what that value added of the paper is. | We apologize for the lack of clarity regarding the paper’s unique contributions, which we failed to articulate sufficiently in the current version. The core value added of this study lies in three interconnected dimensions, which we will emphasize more explicitly in the revised manuscript:  Theoretical Contribution: Existing research on technical vocational education (TVET) quality tends to focus on single-factor analysis (e.g., faculty quality or funding input) or comparative studies of national policies, with limited attention to the configurational mechanisms driving high-quality development. This study pioneers the application of fuzzy-set qualitative comparative analysis (fsQCA) to explore how multiple factors (urban globalization level, industry-education integration, social service capacity, etc.) interact synergistically to shape educational quality—a perspective rarely adopted in TVET research (as supported by our updated literature review, which identifies only 3 out of 87 recent studies using configurational methods).  Methodological Innovation: By integrating Guangdong’s advanced TVET evaluation standards (validated for their industry relevance and policy recognition) with stratified sampling across diverse globalization-level cities, we establish a more nuanced framework for measuring "educational quality" that bridges regional diversity and standardization. This approach addresses the long-standing trade-off between local specificity and generalizability in TVET evaluation research.  Practical Implications: The study identifies three key configurational paths to high-quality TVET development (e.g., "industry-driven + high globalization" and "social service-oriented + regional integration"), providing actionable insights for policymakers designing differentiated support strategies and for colleges seeking to enhance their competitiveness in alignment with urban industrial needs. These findings directly respond to China’s recent National Vocational Education Reform Implementation Plan (2022) call for evidence-based quality improvement.  We will restructure the introduction and discussion sections to foreground these contributions, explicitly contrasting our work with prior studies to highlight the gaps we fill. |
| --- | --- |
| 2. General comment: the paper basically should be in large parts rewritten to make a good and interesting reading. | hank you very much for pointing out the critical issue that "the paper needs to be largely rewritten to improve readability and appeal." Your feedback directly addresses the core shortcomings of the current manuscript, providing us with a clear direction for improvement, and we deeply agree with this and express our sincere gratitude.  We fully understand that the current manuscript has obvious flaws in narrative logic, language expression, and content presentation, failing to meet the academic writing standards of being "fluent and engaging." This reflects that there is still much room for improvement in our academic writing skills, and we sincerely apologize for this.  To address this issue, we have reorganized the logical framework of the entire text, reconstructing the narrative structure around the main line of "research questions — theoretical basis — methodological design — key findings — practical implications" to ensure that each chapter is naturally connected and progresses layer by layer. We will significantly streamline redundant expressions, remove obscure terminology, and simplify the explanation of methodological sections (such as the fsQCA operation process and sample selection logic) using a combination of textual descriptions and visual diagrams. Additionally, we will invite colleagues with rich experience in academic writing to independently review the revised manuscript, focusing on optimizing the accuracy and fluency of language expression.  We will take this revision as an opportunity to profoundly reflect on the shortcomings in our writing. In future research, we will pay more attention to the clarity of academic expression and the appeal of narratives, and continuously improve our writing skills.  Thank you again for your patient guidance. |
| 3. The Introduction is still very superficial. It lacks an indication what data and method will be used in the paper, what value added the paper has and what its structure is. | Sincerely thank you for your comment.  1. Add the following sentence at Line 83-86 “However, most research remains focused on macro-level policy frameworks or single-case analyses, with limited exploration of how these strategies can be empirically validated and adapted to diverse regional contexts in China.” This addition serves to highlight the critical research gap in existing literature: while prior studies have identified key strategies for enhancing TVET quality, they lack systematic empirical validation of how these strategies operate in practice, especially across China’s diverse regional contexts. By explicitly addressing this limitation, we aim to justify our choice of research design—specifically, the use of fuzzy-set qualitative comparative analysis (fsQCA) on developmental data from 16 technical colleges. This method is uniquely suited to explore combinatorial effects of multiple factors and their contextual adaptability, filling the gap identified in the revised statement.  2. The value of this study is reflected in three dimensions: At the theoretical level, it breaks through the limitations of traditional single-factor analysis, reveals the "social service-driven" mechanism for the high-quality development of TVET from a configurational perspective, and enriches the theoretical framework for improving the quality of vocational education; At the practical level, it identifies key pathways such as "participating in government-funded vocational training programs" and provides operable empirical evidence for institutions to optimize their development strategies; At the methodological level, it verifies the applicability of fsQCA in the field of vocational education and offers a transferable methodological reference for similar global studies (especially meaningful for the configurational analysis of TVET institutions in developing countries).  3. The structure of this paper is arranged as follows: The second part sorts out the relevant theories and existing studies on TVET quality improvement, and clarifies the research gaps; The third part elaborates on the research design, including case selection criteria, data sources, variable measurement, and the fsQCA analysis process; The fourth part presents the results of the fsQCA analysis and explains the key configurations and bottleneck types of high-quality development; The fifth part discusses the theoretical connotations and practical implications of the research findings, responding to the practical needs of global TVET development; The sixth part summarizes the research limitations and future research directions. |
| 4. The Literature Review is superficial too. It is difficult to understand and simply boring. | Thank you for your valuable feedback. We take seriously your comment that "The Literature Review is superficial too. It is difficult to understand and simply boring." We have thoroughly rewritten and revised the literature review section to address these concerns.  The specific revisions can be found in lines 89–117 and 142–146 of the manuscript. Key improvements made during the revision include:  Strengthening the logical structure: The content is reorganized around the framework of "theoretical foundations—current research progress—research gaps," with clear hierarchical relationships to enhance readability.  Deepening analytical depth: We added critical evaluations of core literature (rather than mere listing) to clarify the contributions and limitations of existing studies.  Improving expression: Reduced the overuse of obscure jargon and added explanations linking theory to practice to enhance comprehensibility.  Highlighting relevance to the study: Emphasized the logical connections between the literature review, our research questions, methodology, and core hypotheses to strengthen the review’s relevance. |
| 5.The sample description of on page 14 is still very superficial and the reasons for choosing 40 cases of outstanding technician colleges remains still unclear.  6.A similar comment concerns the 16 technician colleges selected as the final research objects. | Thank you for your suggestion. We have carefully revised the sample selection section to address this concern, with specific improvements detailed below.  In response to your feedback, we have supplemented the sample description and added a flowchart (see Figure 2) to visually illustrate the screening process, ensuring transparency in how the 16 research samples were selected from the initial database. The revised content can be found in lines 343-351 of the manuscript, with the new flowchart inserted on page 14.  Key revisions include:  1. Clarifying the source of the 40 colleges: We explicitly explain that the 40 excellent technical vocational colleges are derived from Yang Shengwen’s (2019) case database [1], which systematically documents institutions recognized for their developmental practices in China’s TVET sector, providing a reliable baseline for sample selection.  2. Detailing the stratified sampling logic: The flowchart outlines the multi-step screening process.  3. Justifying the focus on "excellent but non-qualified" colleges: We clarify that these 40 colleges were categorized as "excellent" based on industry recognition but "non-qualified" under Guangdong’s high-level standards, making them suitable for comparing factors distinguishing high-quality vs. sub-optimal development—a critical contrast for our fsQCA analysis. |
