# Supplementary material for: How to promote the quality of technical education? A fuzzy-set qualitative analysis of 16 technical colleges in China
Source: PLoS One. 2025 Oct 6;20(10):e0332115. doi: 10.1371/journal.pone.0332115 (PMC12500140; doi:10.1371/journal.pone.0332115)
Supplement: Attachment — Submitted filename: Response_to_Reviewers_auresp_3.docx [file pone.0332115.s008.docx]

Rebuttal letter

Dear Editor and Reviewers,

Thank you for giving us the opportunity to submit a revised draft of the manuscript “How to Promote the Quality of Technical Education？A Fuzzy-set Qualitative Analysis of 16 Technical Colleges in China” for publication in the Journal of PLOS ONE. We appreciate the time and effort that you and the reviewers dedicated to providing feedback on our manuscript and are grateful for the insightful comments on and valuable improvements to our paper.

First, we would like to specifically acknowledge the meticulous and professional feedback on the Introduction section, which has provided us with critical insights to address its previously identified limitations. Your pointed observations helped us clearly recognize gaps in the original draft, including the lack of explicit articulation of research data sources, methodological rationale, the theoretical and practical value of the study, and the manuscript’s structural roadmap. Guided by your comments, we have refined the Introduction to enhance its depth, coherence, and academic rigor—not only to address these gaps but also to better contextualize the study within existing literature and highlight its unique contributions. This revision process has not only improved the manuscript itself but also deepened our understanding of how to construct a logically rigorous and compelling academic introduction, for which we are truly thankful.

We have carefully checked the revised Introduction to ensure that all the key information you noted (data, methods, study value, structure) is fully and organically integrated, rather than superficially added. This revision not only makes the Introduction more comprehensive and rigorous but also helps readers quickly grasp the core logic and contributions of the study—directly addressing the “superficiality” issue you identified.

Once again, we would like to thank you for your patient guidance and valuable insights. Your comments have been pivotal to improving the rigor and clarity of our manuscript. We have attached the revised manuscript for your review and welcome any further suggestions you may have. We remain committed to making additional refinements to meet the high standards of PLOS ONE.

Sincerely,

Fan Zhang

Department of Education/Shaanxi Normal University/Xi’an/Shaanxi Province/China

**Reply to comments from academic editors**

Reviewer #7

| 1.The Introduction is still very superficial and besides one new sentence nothing has changed in the Introduction. It still lacks an indication what data and method will be used in the paper, what value added the paper has and what its structure is. | Thank you for this precise feedback. We have completely rewritten the Introduction(lines 54–113) to address each point: a new paragraph is added to state "Taking 16 Chinese technical colleges as cases, we draw on administrative data—annual institutional quality reports and provincial TVET bulletins—and apply fsQCA to uncover equifinal pathways to high-quality TVET development"; a "Research Contributions" subsection is added to clarify the value in three aspects (theory: the first configurational study of Chinese technician colleges, revealing a "social-service-driven" mechanism; practice: identifying actionable levers like participation in government-funded training programmes; method: demonstrating the transferability of fsQCA to TVET research in emerging economies); and the conclusion includes a numbered roadmap (1. Theoretical Foundations and Gaps; 2. Research Design; 3. fsQCA Results; 4. Implications; 5. Limitations and Future Directions) to preview the structure. The revised Introduction now fully meets the required depth and clarity. |
| --- | --- |
| 2. I totally don’t understand why the authors mention those issues in their reply to my comment e.g. “The value of this study is reflected in three dimensions: At the theoretical level, it breaks through the limitations of traditional single-factor analysis, reveals the "social service-driven" mechanism for the high-quality development of TVET from a configurational perspective, and enriches the theoretical framework for improving the quality of vocational education; At the practical level, it identifies key pathways such as "participating in government-funded vocational training programs" and provides operable empirical evidence for institutions to optimize their development strategies; At the methodological level, it verifies the applicability of fsQCA in the field of vocational education and offers a transferable methodological reference for similar global studies (especially meaningful for the configurational analysis of TVET institutions in developing countries).” | Thank you for highlighting the lack of an explicit statement of the paper’s contribution. We acknowledge our previous misunderstanding: we had only listed these points in the cover letter instead of incorporating them into the manuscript.  In this revision, we explicitly present the study’s value-added in three dimensions:  •Theory(line57-61):  As a core pillar of global education reform, TVET quality improvement is not only tied to regional industrial upgrading but also characterized by its distinctive social-service function—TVET providers offer publicly accessible vocational-upgrading courses that feed directly back into curriculum innovation, creating a dynamic loop between skill training and educational optimization.  •Practice(line79-83):  These challenges are compounded by China’s dual pressure: emerging sectors like smart manufacturing and the digital economy face pronounced skill shortages, while traditional industries demand reskilling for incumbent workers—making the need to address TVET development bottlenecks even more urgent.  •Method(line96-107):  Fuzzy-set Qualitative Comparative Analysis (fsQCA) is well-suited to address this limitation, as it enables us to not only identify equifinal pathways for TVET high-quality development (given the complex, multi-condition interactive nature of TVET development, where different condition combinations can lead to the same high-quality outcome) but also summarize typical bottleneck types through the analysis of condition necessity and sufficiency (by distinguishing between core conditions that are indispensable for high-quality development and limiting factors whose absence hinders progress; Fiss, 2011). Against this backdrop, the present study builds upon quality cultivation theory while incorporating distinctive features of vocational training to propose an influential factors model for technical colleges' quality development. Taking 16 Chinese technical colleges as research objects, this study utilizes administrative data sources—including institutional annual quality reports and local government TVET development bulletins—to empirically examine these factors. |
| 3. The structure of this paper is arranged as follows: The second part sorts out the relevant theories and existing studies on TVET quality improvement, and clarifies the research gaps; The third part elaborates on the research design, including case selection criteria, data sources, variable measurement, and the fsQCA analysis process; The fourth part presents the results of the fsQCA analysis and explains the key configurations and bottleneck types of high-quality development; The fifth part discusses the theoretical connotations and practical implications of the research findings, responding to the practical needs of global TVET development; The sixth part summarizes the research limitations and future research directions” but fail to include them in the Introduction. | We apologize for the oversight in the previous draft. In the present revision we have inserted an explicit roadmap at the very end of the Introduction (lines 107–113). The paragraph reads:  The paper proceeds systematically: (1) synthesizing theoretical foundations and existing TVET quality improvement literature to delineate research gaps; (2) detailing the research design, encompassing case selection protocols, data sources, variable operationalization, and fsQCA methodology; (3) presenting fsQCA results that reveal key configuration pathways and bottleneck constraints in quality development; (4) discussing theoretical contributions and practical implications aligned with global TVET development needs; and (5) concluding with study limitations and future research directions. |
